# EZH2 enhances PCV2 replication through inhibition of MMP1 and MMP12 transcription activity

**DOI:** 10.1186/s13567-026-01773-3

**Published:** 2026-06-08

**Authors:** Yiyi Shan, Wen Feng, Yueqing Hu, Xiaomei Du, Yue Cao, Yanan Cao, Haifei Wang, Shuai Zhang, Wenbin Bao

**Affiliations:** 1 Key Laboratory for Animal Genetics, Breeding, Reproduction and Molecular Design of Jiangsu Province, College of Animal Science and Technology, Yangzhou University, Yangzhou, 225009 China; 2 College of Life Sciences, Yulin University, Shaanxi, 719000 China

**Keywords:** PCV2, EZH2, MMP1, MMP12

## Abstract

Porcine circovirus type 2 (PCV2), a single-stranded circular DNA virus, primarily targets the porcine immune system. It induces immunosuppression, subsequent porcine circovirus disease (PCVAD), causing substantial economic losses to the global swine industry. Enhancer of zeste homolog 2 (EZH2), a critical member of the polycomb group (PcG) protein family with histone methyltransferase activity, plays a significant role in epigenetic regulation. While previous studies have established EZH2’s role in viral infection and innate immune responses, its function in PCV2 infection remains unclear. In this study, we found that *EZH2* overexpression promoted PCV2 infection, whereas *EZH2* knockdown via specific small interfering RNA (siRNA) and inhibition of its activity reduced PCV2 infection. Furthermore, we identified EZH2-regulated downstream genes by RNA sequencing (RNA-seq) and found that MMP1 and MMP12 were significantly upregulated when EZH2 methyltransferase activity was inhibited by GSK126. Mechanistically, *EZH2* overexpression repressed the promoters of *MMP1* and *MMP12*, thereby facilitating PCV2 replication. Conversely, upon depletion of the *EZH2* methyltransferase domain, inhibition of *MMP1* and *MMP12* promoters is lifted, thereby enabling MMP1 and MMP12 to suppress PCV2 replication. Our findings establish a link between the histone methyltransferase EZH2 and the PCV2 life cycle, advance understanding of the mechanisms underlying PCV2 infection, and suggest that drugs targeting MMP1/MMP12 activity may provide potential therapeutic approaches to prevent and control PCV2 infection and spread.

**Supplementary Information:**

The online version contains supplementary material available at 10.1186/s13567-026-01773-3.

## Introduction

Porcine circovirus is a small, non-enveloped, single-stranded circular DNA virus [1]. After infection, it primarily dysregulates the cytokine profile of the immune response in pigs, resulting in impaired immune cell function, immunosuppression, and depletion of porcine lymphocyte [2], which subsequently leads to porcine circovirus-associated disease (PCVAD). Porcine circovirus is primarily classified into four genotypes: PCV1, PCV2, PCV3, and PCV4. Among them, PCV2 is the primary pathogen responsible for PCVAD [3], and it is also the genotype that causes the most significant economic losses in the global pig industry [4]. PCV2 is further divided into eight genotypes: PCV2a, PCV2b, PCV2c, PCV2d, PCV2e, PCV2f, PCV2g, and PCV2h. Since 2021, the epidemic strain in China has been identified as PCV2d [5]. In the host, the virus primarily targets a variety of immune cells, including tissue cells, macrophages, monocytes, and dendritic cells [6]. The monocyte/macrophage cell line is the primary target of PCV2, while porcine kidney epithelial cell (PK-15) serves as the proliferative cell for the PCV2. Virus-infected cells typically undergo a series of epigenetic modifications to regulate gene expression, thereby resisting viral invasion. In recent years, significant progress has been made in controlling and preventing PCV2, owing to the large-scale and centralized aquaculture industry and the ongoing development of vaccination technologies. However, the continuous emergence of new viral variants has reduced the efficacy of vaccines. Therefore, from the perspective of the host (pig), identifying new targets for regulating PCV2 infection is of great significance for the accurate and effective prevention, control, and treatment of PCV2-induced diseases.

EZH2 is located on chromosome 7q35 and consists of 20 exons encoding 746 amino acids. EZH2 is the catalytic subunit of polycomb repressive complex 2 (PRC2), which trimethylates histone H3 at lysine 27 (H3K27me3) thereby suppressing histone modification, silencing gene transcription, and altering chromatin structure [7]. EZH2 is not only involved in the epigenetic regulation of cellular genes but has also been reported to play a crucial role in viral infections and the innate immune response. During human papillomavirus (HPV) infection, EZH2 facilitates viral persistence by regulating the cell cycle, suppressing the expression of antiviral genes, and modulating the transcription of HPV genes [8]. Epstein–Barr virus (EBV) can upregulate EZH2 expression. Upon knockout of the *EZH2* gene, viral DNA replication and the production of progeny viral particles are enhanced [9]. EZH2 binds to the virus-induced signaling adapter (VISA) in a methyltransferase-independent manner and disrupts the interaction between VISA and RIG-I, thereby inhibiting the *IFN-β* promoter and NF-κB signaling pathway. This promotes the replication of influenza A virus (IAV) in cells [10]; Respiratory syncytial virus (RSV) and rhinovirus (RV) replicate in the airway epithelium, thereby triggering TGF-β-mediated epithelial–mesenchymal transition (EMT). Under EMT induction, ZEB1 modifies the H3K27me3 mark on IRF1 via EZH2, thereby silencing IRF1 expression [11]. These findings suggest that EZH2 modulates viral infection through a complex regulatory mechanism. However, it remains unclear whether PCV2 is involved in histone methylation.

Matrix metalloproteinases (MMPs) are zinc- and calcium-dependent proteolytic enzymes capable of recognizing and cleaving extracellular proteins, thereby degrading the extracellular matrix. To date, at least 20 distinct types of MMPs [12] have been identified in various cellular compartments, including the cytoplasm, nucleus, and mitochondria. They play a critical role in the pathogenesis of cancer metastasis, tumor migration, inflammatory response, and other processes, either through proteasome-dependent or -independent mechanisms, and exhibit antiviral and antibacterial effects [13]. MMP1 and MMP12 interact with multiple chemokine networks (CCL2-4/CXCL1/8) to regulate NF-κB transcriptional activity [14]. In addition, MMP1 releases precursors of signaling molecules, such as pro-TGF-α, EGF-like ligands, and TGF-β, through the cell surface or extracellular matrix, and is capable of processing various key mediators. These include pro-TNF-α, IL-1β, L-selectin, α1-antiprotease inhibitors, C1q, connective tissue growth factor, and insulin growth factor binding protein 1 and 3[15]. MMP12 exhibits a strong positive correlation with inflammatory intestinal diseases. The AMPK–mTOR signaling pathway regulates MMP12 through glycolysis, and inhibition of MMP12 can suppress IL-6 expression in macrophages [16]. Therefore, MMP1 and MMP12 play critical roles in tissue remodeling and the induction of inflammation and are commonly used as biomarkers for inflammatory diseases. Moreover, MMPs are implicated in the viral infection process as well. Several studies have indicated excessive activation of MMP1 in patients with coronavirus disease 2019 (COVID-19), and MMP1 is strongly correlated with the severity of COVID-19 [17]. In respiratory syncytial virus (RSV) infection, MMP12 is significantly upregulated and is targeted by the SARM–TRIF signaling pathway, leading to IFN-γ-independent airway inflammation [18]. In both lethal and sublethal mouse models of neurotropic mouse hepatitis virus infection, lethal infections result in significantly higher MMP12 expression levels compared with sublethal infections [19]. However, no relevant studies have been conducted on MMP1 and MMP12 in PCV2.

In this study, we investigated the role of EZH2 on PCV2 infection and found that EZH2 is a proviral host factor that impacts viral replication through a mechanism by which its methyltransferase activity inhibits *MMP1* and *MMP12* promoter activity, leasing to transcription repression. Our findings provide insights into the PCV2 pathogenesis, offering a novel theoretical framework for its prevention and treatment.

## Materials and methods

### Reagents and antibodies

Dulbecco’s modified Eagle medium (DMEM) medium (6125349) was purchased from Thermo Fisher Scientific (Waltham, MA, USA); fetal bovine serum (F0193) from Sigma-Aldrich (St. Louis, MO, USA); phosphate-buffered saline (PBS; PB180327) was obtained from Punosai (Wuhan, China); DNA marker (MD114-02) from Tiangen (Beijing, China); cell protein lysate buffer (C1053) from Prielec (Beijing, China); and protease inhibitors (HY-K0010) and GSK126 (HY-13470) from MedChemExpress (Monmouth Junction, NJ, USA). GSK126 was dissolved in dimethyl sulfoxide (DMSO) at the indicated concentrations prior to use. The 5× sodium dodecyl sulfate (SDS)–polyacrylamide gel electrophoresis (PAGE) protein loading buffer (S6512300) was obtained from Yisheng (Shanghai, China). The bicinchoninic acid (BCA) protein concentration assay kit (P0010) was sourced from Biyuntian (Shanghai, China); the CCK-8 assay reagent (BMU106) from Yacoin (Wuhan, China); TRIzol reagent (A7A0882) from Hunan Acorui (Changsha, China); and the HisyGo RT Red SuperMix for qPCR (+ gDNA Wiper) (RT101), dual-luciferase assay kit (DL101), and DNA extraction kit (DC112) were all acquired from Novizan (Nanjing, China). Mouse anti-GAPDH antibody (10028230), mouse anti-Hsp90 antibody (10021374), and rabbit anti-EZH2 antibody (21800-1-AP) were purchased from Three Eagles (Wuhan, China). The 2× SuperStarUniversal SYBR Master Mix (18220/04325), goat anti-mouse IgG (CW0102S), and goat anti-rabbit IgG (CW0103S) were supplied by Kangwei Century (Beijing, China), and rabbit anti-PCV2-Cap antibody (GTX641316) was obtained from GeneTex (Irvine, TX, USA).

### Cells and viruses

Porcine kidney cells PK-15 (ATCC, CCL-33) were cultured in high-glucose DMEM medium containing 10% fetal bovine serum and 1% penicillin–streptomycin mixture (100 µg/mL penicillin and 100 µg/mL streptomycin) at 37 °C in a humidified atmosphere containing 5% CO_2_. The PCV2d virus strain used in this study was maintained in our laboratory.

### Total RNA extraction, complementary DNA (cDNA) synthesis, and quantification

Total cellular RNA was extracted using the TRIzol reagent method. RNA integrity was verified by electrophoresis on a 1% agarose gel, and the concentration was quantified using a NanoDrop One spectrophotometer. Complementary DNA (cDNA) was synthesized from the mRNA templates using a commercial reverse transcription kit. Real-time polymerase chain reaction (PCR) analysis was performed using a StepOnePlus real-time PCR system (Thermo Fisher Scientific). The relative messenger RNA (mRNA) expression levels were calculated using the 2^−ΔΔCt^ method, with *GAPDH* serving as the reference gene. The sequences of the primers used are presented in Table 1.

**Table 1:** **Primer sequences for qPCR**

Primer name	Primer sequence (5′ → 3′)	Product size (base pairs [bp])
PCV2-Pig-qPCR-F PCV2-Pig-qPCR-R EZH2-Pig-qPCR-F EZH2-Pig-qPCR-R GAPDH-Pig-qPCR-F GAPDH-Pig-qPCR-R MMP1-Pig-qPCR-F MMP1-Pig-qPCR-R MMP12-Pig-qPCR-F MMP12-Pig-qPCR-R SMAD9-Pig-qPCR-F SMAD9-Pig-qPCR-R AIF1L-Pig-qPCR-F AIF1L-Pig-qPCR-R HABP2-Pig-qPCR-F HABP2-Pig-qPCR-R SLC2A2-Pig-qPCR-F SLC2A2-Pig-qPCR-R HTRA3-Pig-qPCR-F HTRA3-Pig-qPCR-R VTCN1-Pig-qPCR-F VTCN1-Pig-qPCR-R VTCN1-Pig-qPCR-F VTCN1-Pig-qPCR-R	GTTTACATAGGGGTCATAG TGTGCCCTTTGAATACTAC CGATGATGATGACGATGATG CTTCCGCTTGTAAGTATTGG ACATCATCCCTGCTTCTACTGG CTCGGACGCCTGCTTCAC CTCCGAGGGTCAAGCAGACATAATG CTCCGAGGGTCAAGCAGACATAATG TGGGCTGACGATAGAAACAACGCCAATGAC ATGAACATCGGGCACTCCACATCGAGGTT GTGCTCCGTCGCCTACTATGAA CACACTCTGCGTAGACCTCTCC CGCTCAGCAACAGGTTCCAA CGGCAGGTTCTCTTCATCACT TGAGAATTACAACCAGGAGGAGAAC AGGCACAGCGGTAGTAAGGAG TTCAGGAAGAGGCATATCAGGACTC AGCAGATAGACCAAGCAGGATGTG GGTCATTGGCATCAACACGCTCAA ACTTGGTGTGATTGTCCGCATTCG GCAACTCACAGATGCTGGTACATAT CTGGCGTTAGAGTCCACATTCAC GGATTGAGGACTCGGCTGACTT TACTCGTCTGCTGTGATGGATTCTC	148 379 188 207 172 230 128 266 214 157 126 112

### Cellular protein extraction and quantification

Cells were lysed using a lysis buffer supplemented with protease inhibitors. The lysates were then centrifuged at 12 000^rpm^ for 25 min at 4 °C, and the supernatant containing the total protein fraction was collected. The protein concentration was determined using a BCA Protein assay kit according to the manufacturer’s instructions. Subsequently, the protein concentrations were normalized with PBS, and the samples were mixed with 5× SDS–PAGE loading buffer prior to denaturation. Proteins were separated by sodium dodecyl sulfate–polyacrylamide gel electrophoresis (SDS–PAGE) on 10% gel and subsequently transferred onto polyvinylidene (PVDF) membranes. Following transfer, the membranes were blocked and then incubated with specific primary antibodies overnight at 4 °C. After washing with Tris-buffered saline with Tween 20 (TBST), the membranes were incubated with the appropriate horseradish peroxidase (HRP)-conjugated secondary antibodies for 2 h at room temperature and then incubated with an enhanced chemiluminescence (ECL) luminescence solution for exposure development. Protein band intensities were quantified using ImageJ software (National Institutes of Health, Bethesda, Maryland, USA).

### Viral DNA extraction

Viral DNA was extracted following the manufacturer’s instructions for the DNA extraction kit. Cells were harvested and enzymatically lysed by the addition of Proteinase K and Buffer BCL. Absolute ethanol was added to the digested cells to adjust the column environment, followed by shaking and purification through the column. The mixture was transferred to the adsorption column and centrifuged at 12 000 rpm for 1 min. The filtrate was discarded, and the protein and other impurities were removed by the addition of Buffer WA. After discarding the filtrate by centrifugation, salt ions were removed twice by the adding Buffer WB. The residual ethanol was eliminated by centrifugation in empty tubes, and DNA was eluted from the column using the eluate.

### CCK-8 assay

PK-15 cells were seeded into 96-well plates and cultured to a confluence of 60% in high-glucose DMEM medium supplemented with 10% fetal bovine serum in a cell incubator at 37 °C with 5% CO_2_. Different concentrations of GSK126 were applied to each group, and incubation continued for 48 h. CCK-8 reagent was then added, and a blank control was established concurrently. After 1 h of incubation, the absorbance was measured at a wavelength of 450 nm using a microplate reader.

### Synthesis of siRNA and construction of overexpression plasmid and its mutant

siRNA targeting *EZH2* and negative control siRNA were synthesized by Suzhou Gemma Gene Co., Ltd. The siRNAs sequences are provided in Additional file 1. *MMP1* and *MMP12* overexpression plasmids containing FLAG tags were synthesized by Wuhan Jinkarui Biological Engineering Co., Ltd. Luciferase reporter plasmids targeting the promoter regions of *MMP1* and *MMP12* were constructed using the pGL3 basic plasmid vector. The *EZH2* overexpressing plasmid and its mutant (lacking the SET domain) were constructed using the PCDNA3.1 vector. All the synthesized vectors were verified by Sanger sequencing. Plasmids and siRNAs were transfected according to the polyplus jetPRIME^®^ Transfection reagent instructions.

### Dual-luciferase activity assay

293T cells were seeded in 48-well plates and transfected with firefly and Renilla luciferase reporter plasmids using polyplus jetPRIME^®^ reagent, with empty plasmids transfected to ensure equal DNA quantities per well. After 6 h of transfection, GSK126 was added at a concentration of 10 µM, and incubation continued for 36 h. The cells were lysed, collected, and centrifuged at room temperature for 2 min at 11 200 rpm. The supernatant was transferred to the wells of a microplate, and luciferase activity was measured using the dual-luciferase assay kit. Firefly luciferase activity was normalized to Renilla luciferase activity to correct for transfection efficiency.

### Transcriptome sequencing

PK-15 cells were collected from the PCV2 group (PCV2, *n* = 3) and the PCV2 + GSK126 group (PCV2 + GSK126, *n* = 3). Total RNA was extracted and its quality was assessed by electrophoresis on denaturing agarose gels containing 1% formaldehyde. RNA concentration was measured using a NanoDrop One nucleic acid/protein concentration analyzer. RNA was reverse transcribed into cDNA and sequenced on an Illumina Hiseq 2500 platform at Oebiotech (Shanghai). The corrected *p*-value < 0.05 and |log_2_ (fold change)|< 1 were considered as the identification principles of differentially expressed genes (DEGs). GOseq was used to annotate the Gene Ontology (GO) functions of DEGs in the resulting data. The enrichment of DEGs in Kyoto Encyclopedia of Genes and Genomes (KEGG) pathways was analyzed using KOBAS software (version 3.0). GO term and KEGG pathway analyses were performed after applying a cutoff value of 0.05 for statistical correction.

### Chromatin immunoprecipitation‑sequencing (ChIP) assay

PK-15 cells were seeded in 10-cm cell culture dishes and transfected with corresponding plasmids when cell confluency reached 50%. After 48 h of culture, cells were harvested and cross-linked with 1% formaldehyde, followed by glycine buffer to quench the cross-linking reaction. After washing, cell pellets were collected and lysed in lysis buffer containing protease inhibitor cocktail. Lysates were sonicated at low temperature for 3 min 30 s to shear genomic DNA into 200–700 base pairs (bp) fragments. Sonicated supernatants were diluted with ChIP buffer, with an aliquot reserved as input sample. The remaining samples were incubated with 3 μL H3K27me3 antibody or IgG antibody overnight at 4 °C and then mixed with Protein A/G Magnetic Beads for another 2 h incubation. Beads were washed with Wash Buffer, and the bound DNA was eluted and recovered. All samples were subjected to qPCR analysis, with results normalized to input values (percentage input = 2^ (Ct, Input − Ct, IP) × 100. Relative enrichment levels were expressed as fold changes versus IgG control. Primers used for ChIP assays: *MMP1* forward, CCCCAAAGGCAGAAGTTTTATGT; *MMP1* reverse, TTGGAGGAGAGTGCTTTTTCTG; *MMP12* forward, GGTGAAACCATTCAGGAGAAGTG; *MMP12* reverse, AGCTCCAACAGCAGTGACCT.

### Statistics and reproducibility

The 2^−ΔΔCt^ method was used to calculate relative quantitative data, with expression levels normalized to internal reference genes. Statistical analyses were performed using SPSS software version 25.0 (SPSS, Inc., Chicago, Illinois, USA). *P*-values were calculated using unpaired *t*-tests between two groups or one-way analysis of variance (ANOVA). All data are presented as the mean ± standard deviation (SD) of triplicate samples. A *p*-value of < 0.05 was considered statistically significant. Statistical methods were not used to predetermine the sample size, and no data were excluded from the analysis.

## Results

### PCV2 infection upregulates EZH2 expression in PK-15 cells

**Figure 1:**
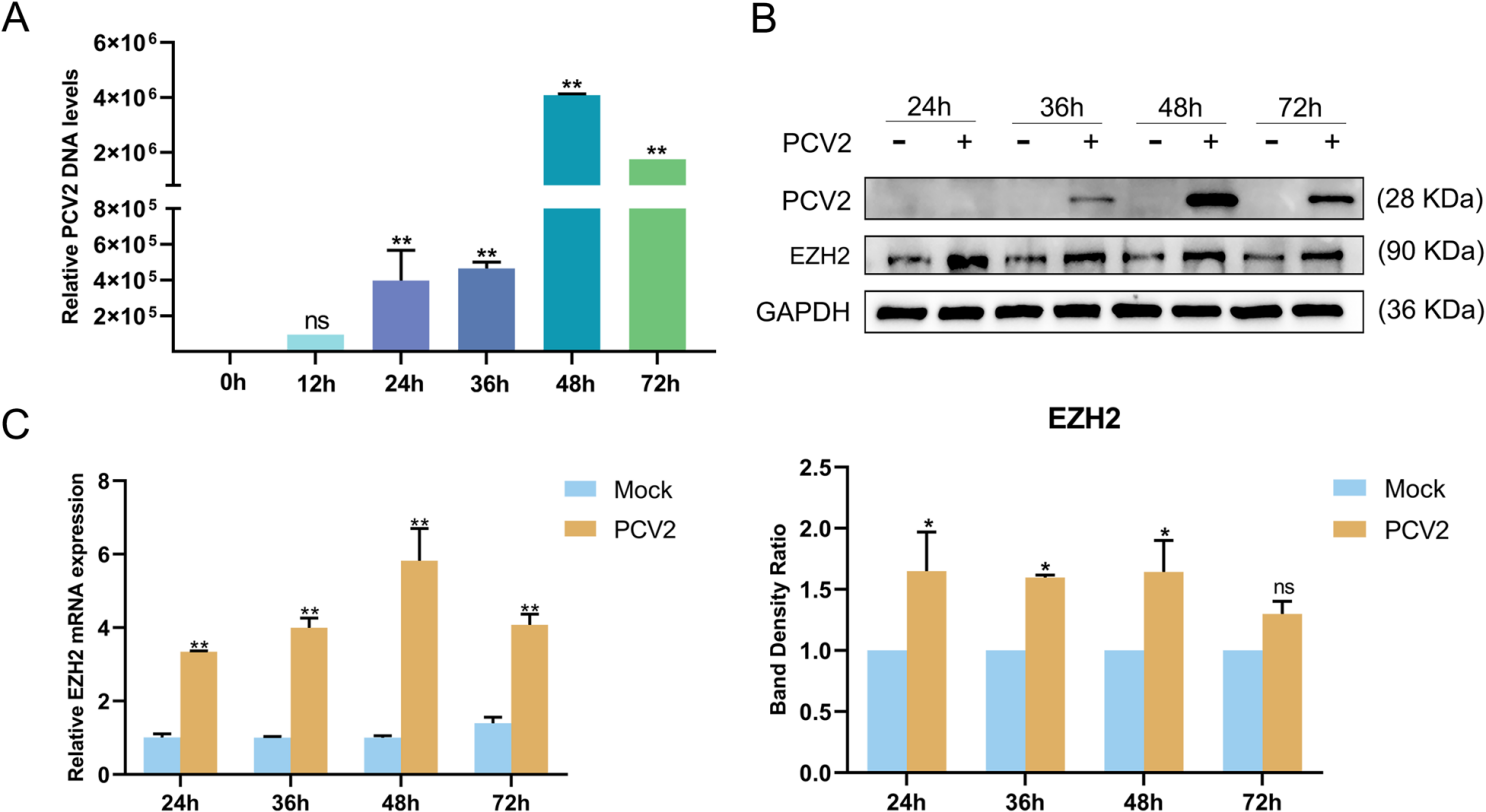
**PCV2 infection increases EZH2 expression in PK-15 cells**. **A** Relative *PCV2* DNA levels in PK-15 cells at 0, 12, 24, 36, 48, and 72 h post infection by qPCR. **B** PCV2-Cap and EZH2 protein expression levels were detected by western blot and calculated with ImageJ. **C** *EZH2* mRNA levels at 24, 36, 48, and 72 h post PCV2 infection were detected by qPCR with reverse transcription (RT–qPCR). Data are presented as means ± standard errors from three independent experiments. *p < 0.05, **p < 0.01, while “ns” indicates no significant difference.

To investigate the effect of PCV2 infection on EZH2 expression in porcine kidney cells, PK-15 cells were infected with PCV2 at a multiplicity of infection (MOI) of 1. Cells were harvested at 0, 12, 24, 36, 48, and 72 h post infection, and the viral replication kinetics were assessed by quantitative real-time PCR and western blot (Figure 1A and B). These analyses revealed PCV2 replication peaked at 48 h post infection. Notably, PCV2 infection markedly upregulated EZH2 expression with a similar temporal pattern to viral replication (Figure 1B and C). These results suggest that EZH2 participates in PCV2 infection.

### EZH2 enhances PCV2 replication in PK-15 cells

**Figure 2:**
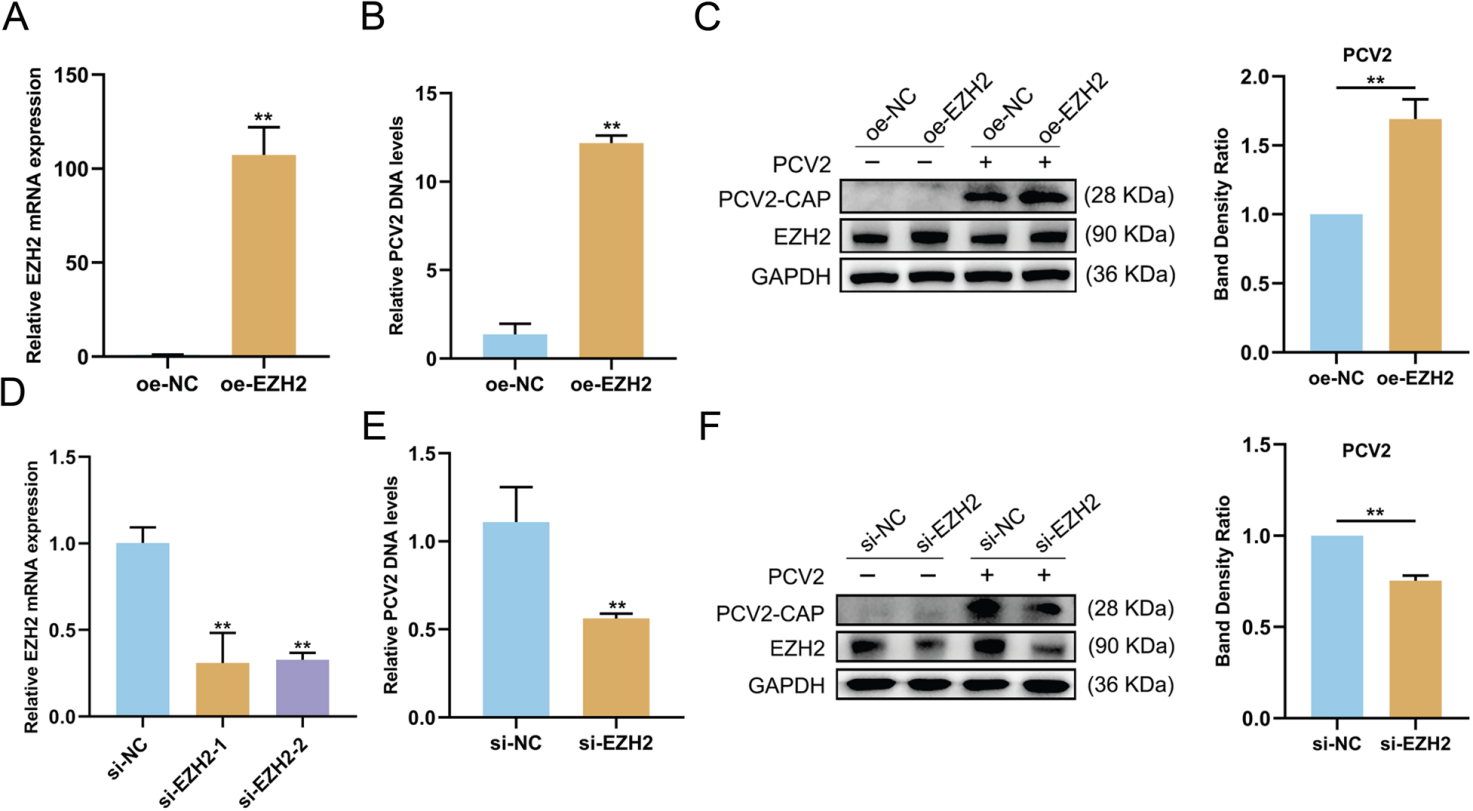
**EZH2 enhances PCV2 replication in PK-15 cells**. **A** The overexpression efficiency of *EZH2* was examined using RT–qPCR. **B**, **C** qPCR and western blot analyses of PCV2 Cap levels in oeEZH2‑transfected PK‑15 cells; band intensities were quantified by ImageJ. **D** The interference efficiency of siEZH2 was detected by RT–qPCR. **E**, **F** PK‑15 cells was transfected with siNC or siEZH2, and PCV2 replication was detected by qPCR and western blot; band intensities were quantified by ImageJ. Data are presented as means ± standard errors from three independent experiments. *p < 0.05, **p < 0.01.

Given that PCV2 infection upregulates *EZH2*, we next examined the role of *EZH2* in PCV2 replication. We constructed an *EZH2* overexpression plasmid and transfected it into PK-15 cells. Transfection of the *EZH2* plasmid significantly upregulated *EZH2* expression, as evidenced by increased *EZH2* mRNA and protein levels (Figure 2A and C). Moreover, *EZH2* overexpression significantly increased PCV2 *Cap* DNA and protein levels (Figure 2B and C).

To further evaluate *EZH2*’s role, we designed two small interfering RNAs (siRNAs) targeting *EZH2*; siEZH2-2 exhibited the strongest silencing efficiency and markedly reduced *EZH2* protein expression (Figure 2D and F). Consistently, *EZH2* knockdown significantly decreased PCV2 replication (Figure 2E and F). These findings indicate that *EZH2* positively regulates PCV2 replication.

### EZH2 promotes the replication of PCV2 through its methyltransferase activity

**Figure 3:**
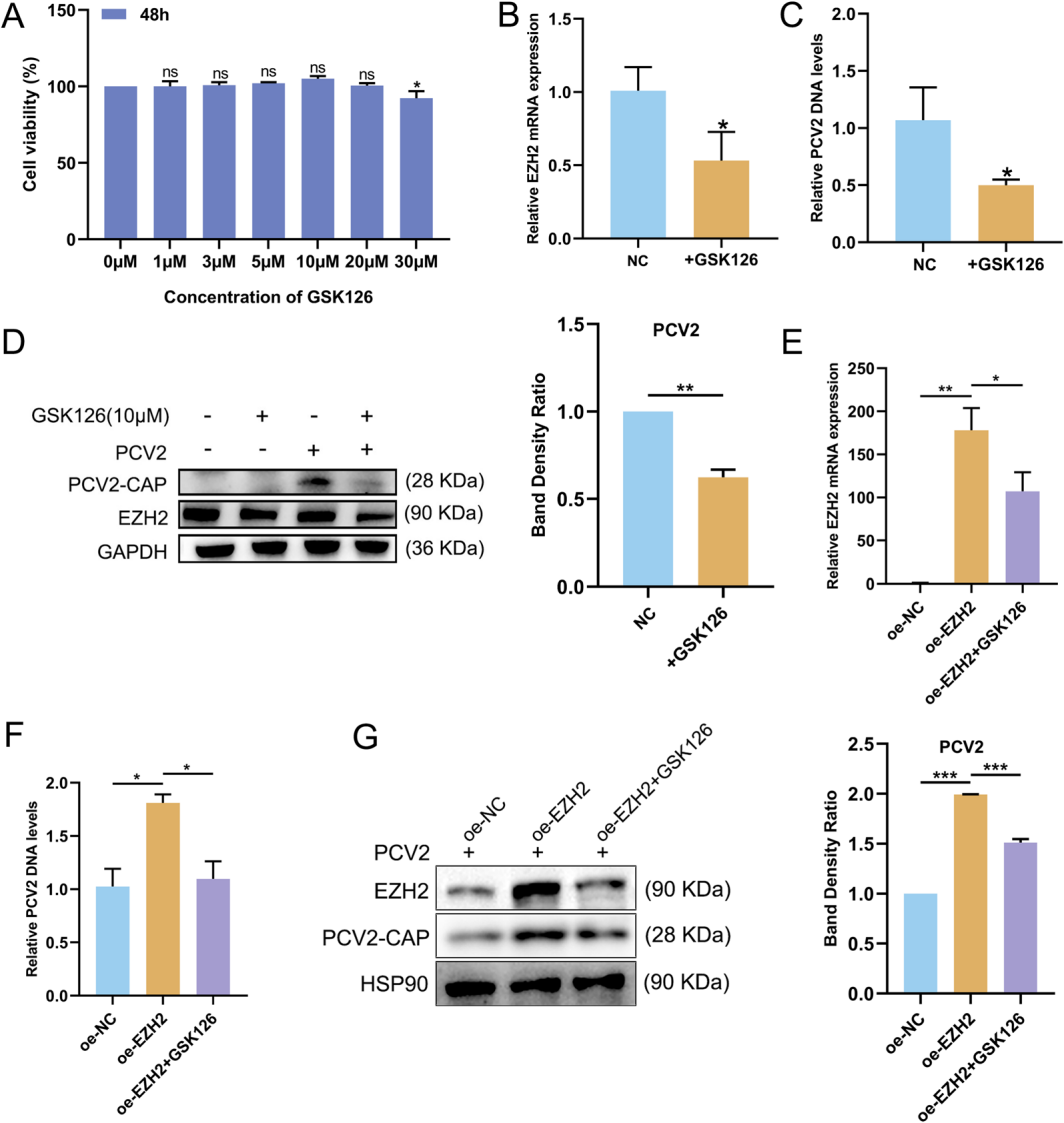
**EZH2 promotes the replication of PCV2 through its methyltransferase activity**. **A** Cell viability under different concentrations of GSK126 was detected by CCK-8 assay for 48 h. **B** *EZH2* mRNA level under GSK126 treatment was detected by RT–qPCR. **C**, **D** PCV2 replication before and after GSK126 treatment was evaluated by qPCR and western blot; band density ratios were quantified. **E** *EZH2* mRNA levels were detected by RT–qPCR before and after GSK126, oeNC, and oeEZH2 treatments. **F**, **G** PCV2 replication before and after GSK126, oeNC, or oeEZH2 treatment was assessed by qPCR and western blot; band density ratios quantified. Data are presented as means ± standard errors derived from three or six (**A**) independent experiments. *p < 0.05, **p < 0.01, while “ns” indicates no significant difference.

GSK126, a specific EZH2 inhibitor, was used to suppress its methyltransferase activity. First, CCK-8 assays assessed cell viability at various GSK126 concentrations, showing no cytotoxicity to cells at concentrations below 20 μM; thus, 10 μM was selected for subsequent experiments (Figure 3A). RT–qPCR confirmed that GSK126 treatment effectively reduced *EZH2* mRNA levels (Figure 3B). To evaluate the effect of GSK126 on PCV2 replication, PK-15 cells were treated with 10 μM GSK126, revealing significantly reduced Cap protein expression (Figure 3C and D). Furthermore, treating *EZH2*-overexpressing cells with GSK126 reduced *EZH2* mRNA levels (Figure 3E). Notably, inhibiting EZH2 activity with GSK126 attenuated the proviral effect of *EZH2* overexpression (Figure 3F and G). These results indicate that EZH2 positively regulates PCV2 replication and that this regulation requires its methyltransferase activity.

### Identification of potential EZH2 downstream targets via RNA-seq

**Figure 4:**
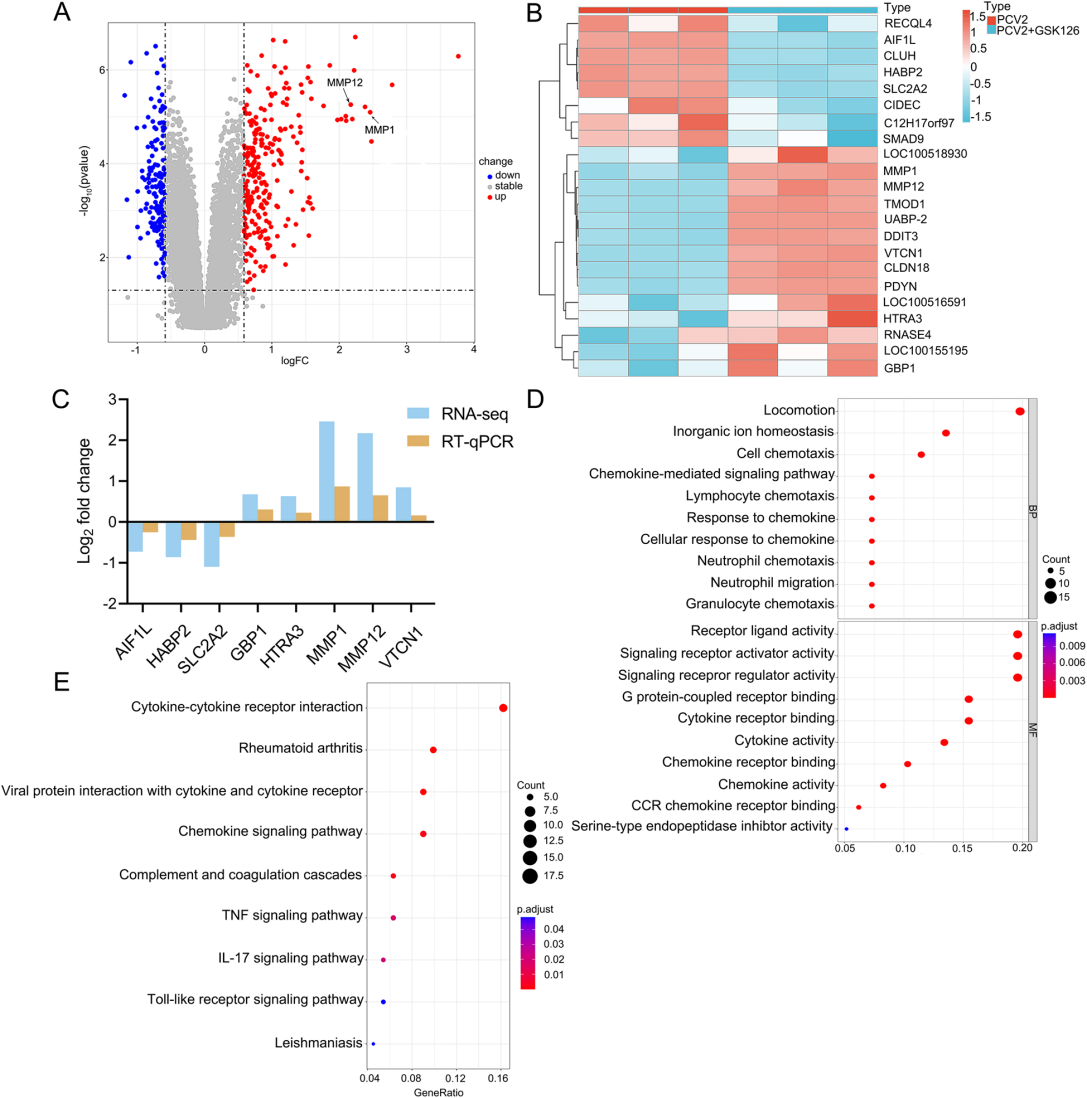
**Identification of potential downstream targets of EZH2**. **A** Volcano plot of differentially expressed genes. Blue plots indicate downregulated genes and red plots indicate upregulated genes. **B** Heat map of differentially expressed genes, where red indicates relatively high expression of protein-coding genes and blue indicates relatively low expression of protein-coding genes. **C** RT–qPCR was used to detect differential gene expression and to verify the sequencing accuracy. **D**, **E** GO and KEGG enrichment analyses. The bubble area represents the number of enriched genes, and the bubble color represents the enrichment salience. Data are presented as means ± standard errors from three independent experiments. *p < 0.05, **p < 0.01.

To clarify the mechanism by which EZH2 regulates PCV2 infection, we performed RNA sequencing (RNA-seq) on PCV2-infected cells and PCV2-infected cells treated with GSK126. A total of 399 differentially expressed genes (DEGs) were identified, including 91 downregulated and 308 upregulated genes (Figure 4A and Additional file 2). The heat map displays significantly upregulated genes, with *MMP1* and *MMP12* being key candidates (Figure 4B). Next, we validated the RNA-seq data via RT–qPCR, randomly selecting eight DEGs (*AIF1L*, *HABP2*, *SLC2A2*, *GBP1*, *HTRA3*, *MMP1*, *MMP12* and *VTCN1*). The results showed that *AIF1L*, *HABP2*, and *SLC2A2* were significantly downregulated, while *GBP1*, *HTRA3*, *MMP1*, *MMP12*, and *VTCN1* were markedly upregulated, which was consistent with the RNA-sequencing results and confirmed the reliability of the data (Figure 4C). GO enrichment analysis (biological process, BP; molecular function, MF) further linked MMP1 and MMP12 to the functional roles of DEGs. The DEGs were significantly enriched in BPs, such as locomotion, inorganic ion homeostasis, and cell chemotaxis, as well as the MF of regulation of receptor–ligand activity. Notably, these enriched functions align with the well-documented roles of MMP1 and MMP12: degrading the extracellular matrix (ECM) and cleaving chemokine precursors to support cell migration; regulating receptor–ligand interactions to modulate signaling cascades; and adjusting ion channels to maintain intracellular and extracellular ion balance (Figure 4D). KEGG enrichment analysis revealed DEGs were enriched in pathways including cytokine–cytokine receptor interaction, rheumatoid arthritis, and viral protein–cytokine receptor signaling. This suggests that under the context of immune dysregulation in GSK126-treated PCV2-infected cells, the binding of cytokines to cell surface receptors may induce *MMP1* and *MMP12* overexpression, which maintains amplified inflammatory signaling (Figure 4E).

### EZH2 inhibits *MMP1* and *MMP12* transcription via its methyltransferase activity

**Figure 5:**
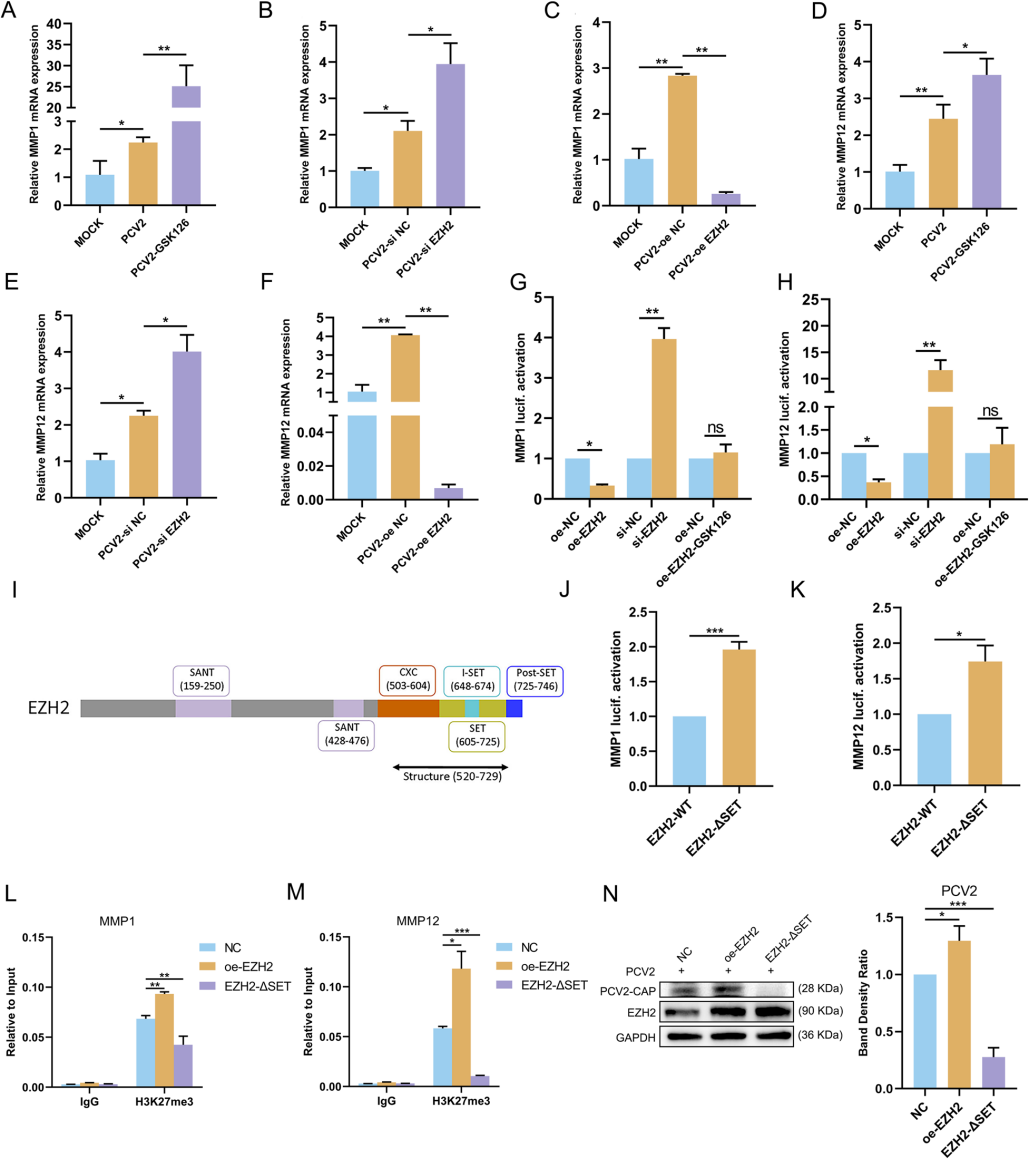
**EZH2 inhibits the transcription of** ***MMP1*** **and** ***MMP12***. **A**–**C** RT–qPCR detected *MMP1* mRNA expression in PCV2-infected PK-15 cells before and after treatment with GSK126, siEZH2, or oeEZH2. **D**–**F** RT–qPCR detected *MMP12* mRNA expression in PCV2-infected PK-15 cells before and after treatment with GSK126, siEZH2, or oeEZH2. **G**, **H** Dual-luciferase reporter was performed to detect the activity of *MMP1* and *MMP12* promoter regions in cells treated with GSK126, or transfected with siEZH2 or oeEZH2. **I** Distribution of functional domains of *EZH2*. **J**, **K** Dual-luciferase reporter assays were conducted to examine *MMP1* and *MMP12* promoter activities following ablation of EZH2 methyltransferase activity. **L**, **M** ChIP assays with anti‑H3K27me3 antibody or IgG control to detect binding regions of *MMP1* and *MMP12* promoters after transfection with oeEZH2 and EZH2-ΔSET plasmids. **N** PCV2 replication before and after oeEZH2 or EZH2-ΔSET treatment was assessed by western blot; band density ratios were quantified. Data are presented as means ± standard errors derived from three or six (G, H, J, K) independent experiments. *p < 0.05, **p < 0.01, while “ns” indicates no significant difference.

MMPs participate in extracellular matrix remodeling, inflammatory mediator modulation, and immune barrier alteration [20, 21] and play crucial roles in physiological processes, pathological mechanisms, and signaling pathways. Since EZH2 represses gene transcription via histone methylation, we hypothesized EZH2 negatively regulates MMP1 and MMP12 expression. First, we confirmed PCV2 infection induces *MMP1* and *MMP12* expression. As expected, *EZH2* knockdown or activity inhibition significantly increased *MMP1* transcript levels, while *EZH2* overexpression caused a significant decrease in *MMP1* levels (Figure 5A–C). *MMP12* exhibited an identical expression pattern (Figure 5D–F), demonstrating EZH2 downregulates *MMP1* and *MMP12* expression. To further verify whether the inhibitory effect of EZH2 on MMP1 and MMP12 was mediated by its histone methyltransferase activity, *MMP1* and *MMP12* promoter were cloned into pGL3 basic plasmid for dual-luciferase reporter assays. The results showed that EZH2 significantly suppressed both *MMP1* and *MMP12* promoter activities. Notably, inhibition of EZH2 methyltransferase function by GSK126 significantly enhanced *MMP1* and *MMP12* promoter activities (Figure 5G and H). Furthermore, on the basis of the genomic localization of distinct functional domains of *EZH2*, we constructed an *EZH2* mutant plasmid lacking the SET domain (EZH2-ΔSET), which abrogates its histone methyltransferase activity (Figure 5I). Our results demonstrated that the loss of EZH2 methyltransferase activity significantly upregulated the promoter activities of *MMP1* and *MMP12* (Figure 5J, K). In addition, compared with the NC group, the enrichment of H3K27me3 on the promoters of *MMP1* and *MMP12* was significantly reduced in the EZH2-ΔSET group (Figure 5L, M), and the viral protein was also significantly decreased (Figure 5N). Collectively, these findings suggest that EZH2 inhibits the transcription of *MMP1* and *MMP12* by regulating their promoter activities through its methyltransferase function.

### EZH2 promotes PCV2 infection by regulating MMP1 and MMP12 expression

**Figure 6:**
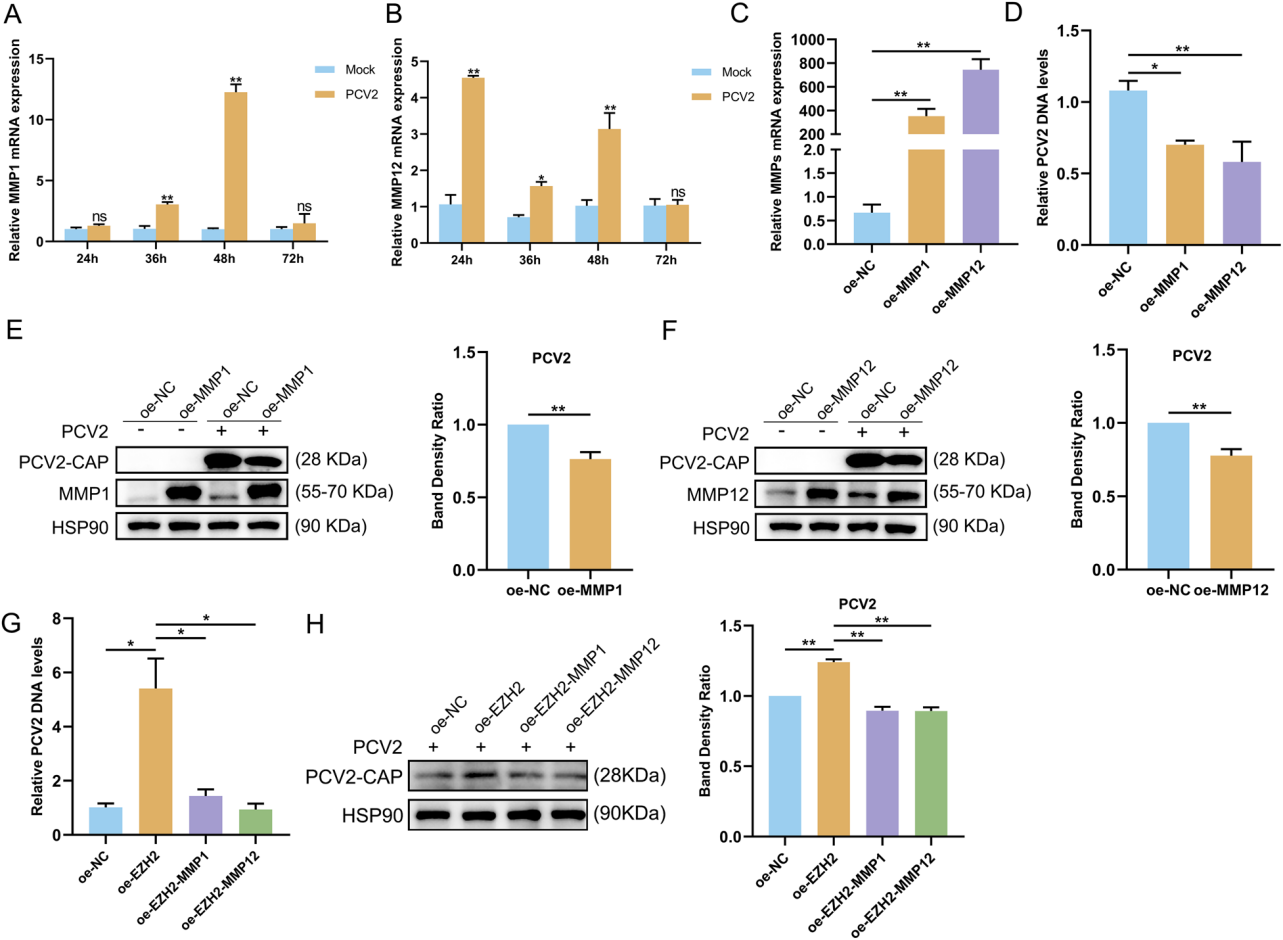
**Overexpression of** ***MMP1*** **and** ***MMP12*** **inhibits PCV2 infection**. **A**, **B** RT–qPCR analyzed *MMP1* and *MMP12* expression at 24, 36, 48, and 72 h. **C** RT–qPCR detected *MMP1* and *MMP12* overexpression efficiency. **D** PCV2 levels in PCV2-infected cells overexpressing *MMP1*/*MMP12* were determined by qPCR **E**, **F** Western blot detection of PCV2 CAP protein in PCV2-infected cells with *MMP1*/*MMP12* overexpression; band intensities were quantified by ImageJ. **G**, **H** qPCR and western blot analyses of PCV2 levels were performed in PCV2-infected cells transfected with oeEZH2, oeMMP1, or oeMMP12; band intensities were quantified using ImageJ. Data are presented as means ± standard errors from three independent experiments. *p < 0.05, **p < 0.01, while “ns” indicates no significant difference.

The above findings showed that EZH2 regulates *MMP1* and *MMP12* transcription via its methyltransferase activity, prompting us to investigate whether the proviral effect of EZH2 is mediated through regulating MMP1 and MMP12 expression. We first examined the temporal expression patterns of MMP1 and MMP12 following PCV2 infection. Consistent with transcriptome sequencing results (Additional file 3), PCV2 infection significantly increased *MMP1* and *MMP12* expression (Figure 6A and B). Next, we constructed eukaryotic expression plasmids encoding *MMP1* and *MMP12* (Figure 6C) to evaluate their effects on PCV2 infection. The results showed that overexpression of either *MMP1* or *MMP12* significantly reduced PCV2 *Cap* gene and protein levels (Figure 6D–F). Furthermore, co-transfecting *MMP1* and *MMP12* overexpression plasmids into *EZH2*-overexpressing cells attenuated *EZH2*-induced enhancement of PCV2 proliferation (Figure 6G and H), Collectively, these findings demonstrate that EZH2 directly regulates MMP1 and MMP12 expression and their upregulation inhibits PCV2 replication in vitro.

## Discussion

Porcine circovirus is widespread globally, with PCV2b and PCV2d being predominant strains in China. ORF1 of PCV2 is located on the positive strand of the genome and is responsible for encoding proteins involved in viral replication (Rep and Rep′). Rep′ is a truncated transcript generated by cleavage [22], which interacts with Rep to form homodimers or heterodimers, playing a crucial role in various stages of viral replication [23]. The PCV2 capsid is composed of 60 protein subunits (predominantly consisting of the Cap protein), forming an icosahedral structure without an envelope [24]. Its structure is relatively stable and resistant to degradation by environmental factors, which contributes to its high transmission efficiency. The high frequency of mutations in the Cap of PCV2 enables rapid adaptation to the host, allowing the virus to evade immune surveillance through alterations in the epitope structure [1]. Currently, there is no approved treatment for PCV2 and the PCV2-induced PCVAD, and vaccine protection alone is insufficient to effectively prevent PCV2 transmission and persistent infection.

Epigenetic therapy represents a novel approach capable of silencing various oncogenes, controlling infections, and combating diverse viruses. EZH2, a core component of the PRC2 that mediates epigenetic modifications, is a key regulator of gene transcriptional repression. Although EZH2 is predominantly studied in the context of human cancers, its role in viral infections has garnered increasing attention in recent years. During EBV infection, the virus upregulates EZH2 expression; however, *EZH2* knockout significantly enhances the expression of EBV during the lytic phase [9]. In the present study, we observed that PCV2 infection also induces EZH2 upregulation in host cells, a finding that prompted our investigation into the mechanistic interplay between EZH2 and PCV2. Notably, our results demonstrated that, unlike in EBV infection, pharmacological inhibition or genetic silencing of *EZH2* significantly suppressed PCV2 proliferation. Further mechanistic analyses revealed that EZH2 physically interacts with MMP1 and MMP12 and catalyzes H3K27 methylation at the promoter regions of *MMP1* and *MMP12* via its intrinsic histone methyltransferase activity. This epigenetic modification consequently inhibited the transcriptional activity of *MMP1* and *MMP12*, thereby facilitating PCV2 infection. Conversely, loss of EZH2 enzymatic activity led to elevated transcriptional activity of *MMP1* and *MMP12* and a concomitant reduction in PCV2 infectivity.

Multiple studies have reported that the EZH2 inhibitor GSK126 can induce an antiviral state and exert potent inhibitory effects on a variety of DNA and RNA viruses, including human cytomegalovirus, adenovirus, herpesvirus, and Zika virus [21]. Herein, we report for the first time the antiviral activity of GSK126 against PCV2, showing that GSK126-mediated EZH2 inhibition significantly impairs PCV2 replication in host cells. EZH2 inhibitors such as GSK126 exert their biological functions by targeting the chromatin domain marked by H3K27me3 to repress gene transcription; they also modulate multiple signaling pathways associated with inflammation, cellular stress, and antipathogen immunity and promote immune cell trafficking to sites of infection, collectively contributing to robust antiviral and immune responses. These properties highlight the great potential of EZH2 inhibitors as broad-spectrum antiviral agents for clinical development [21].

Accumulating evidence supports the epigenetic regulation of MMP1 and MMP12 expression in mammalian cells. For instance, proMMP1 secretion is epigenetically controlled, and treatment with the DNA methyltransferase inhibitor 5-aza-2′-deoxycytidine (5-aza-dC), either alone or in combination with trichostatin A (TSA), reduces proMMP1 protein expression in human fibrosarcoma cell lines [25]. Consistent with these findings, our study identified EZH2 as a negative regulator of MMP1 and MMP12 expression, as GSK126-mediated EZH2 inhibition significantly upregulated the transcription of both genes. According to previous reports, the functional domains of *EZH2* include the WD-40 binding domain (WDB), domain I, and domain II, which are primarily involved in the assembly of the PRC2 complex. In addition, two SANT domains mediate interactions between chromatin-remodeling factors and histones. Other critical domains consist of the cysteine-rich (CXC) domain and the SET domain. The SET domain, an evolutionarily conserved catalytic domain located at the C terminus, is responsible for the histone methyltransferase activity of EZH2 [26]. On this basis, we constructed a truncated *EZH2* mutant plasmid lacking the SET domain (EZH2-ΔSET). Dual-luciferase reporter and ChIP assays confirmed that EZH2 exerts its repressive effect *MMP1* and *MMP12* transcription through its SET domain-dependent methyltransferase activity, which drives H3K27 methylation at the promoters of these two genes. Notably, a previous study reported that ultraviolet (UV) irradiation induces EZH2 expression, and the EZH2/P65/P50 complex is recruited to the *MMP1* promoter to enhance its transcriptional activity in a methyltransferase activity-independent manner [26]. These studies reveal that EZH2 enhances MMP1 expression independently of its methyltransferase activity, highlighting the functional versatility of this protein.

MMPs have been well characterized as important host antiviral factors during viral infection. For example, macrophage secreted MMP12 drives *NFKBIA* transcription upon coxsackievirus or respiratory syncytial virus (RSV) infection, thereby promoting interferon-α (IFN-α) secretion and exerting a host-protective effect. Moreover, in the setting of hyperactivated immunity, extracellular MMP12 cleaves the binding site of the type I IFN receptor 2 (IFNAR2), thereby attenuating excessive immune responses and preventing immunopathology. [27]. Consistent with these reports, our study demonstrated that PCV2 infection induces MMP1 and MMP12 expression in porcine kidney (PK15) cells, and ectopic overexpression of either *MMP1* and *MMP12* markedly inhibited PCV2 proliferation. We hypothesize that PCV2 infection triggers an innate antiviral response in host cells, leading to the induction of MMP1 and MMP12; activated MMP1 and MMP12 then initiate downstream immune signaling cascades and establish a positive feedback loop to restrict viral spread. This mechanism may underpin the observed upregulation of MMP1 and MMP12 following PCV2 infection. While our study confirmed a close functional interaction between EZH2 and MMP1/MMP12 in modulating PCV2 proliferation, whether the dynamic expression of MMP1 and MMP12 is integrated into the host innate immune signaling network during PCV2 infection remains to be further investigated.

In this study, we demonstrate that EZH2 enhances PCV2 replication by repressing the transcription of *MMP1* and *MMP12* via its histone methyltransferase activity. Specifically, *EZH2* overexpression promotes PCV2 replication in PK-15 cells, while *EZH2* knockdown via specific siRNA or activity inhibition using GSK126 significantly reduces viral replication. Transcriptomic analysis identified MMP1 and MMP12 as key downstream targets negatively regulated by EZH2. Mechanistically, EZH2 suppresses the promoter activity of these two genes, and overexpression of either *MMP1* or *MMP12* markedly inhibits PCV2 replication. Collectively, these results reveal that EZH2 acts as a proviral host factor that facilitates PCV2 infection by epigenetically silencing the antiviral genes *MMP1* and *MMP12*. This study provides insight into the mechanisms of PCV2 infection and provides a theoretical foundation for developing drugs targeting MMP1 and MMP12 activity to prevent and control PCV2 infection and transmission.

## Supplementary Information

**Additional file 1. SiRNAs sequences.**

**Additional file 2. Combined analysis of KEGG enriched differential genes (PCV2 VS PCV2 + GSK126).**

**Additional file 3. Combined analysis of KEGG enriched differential genes (PCV2 VS NC).**

## Data Availability

All of the data analyzed in this work are included in this published article. The raw data generated in this study are available upon reasonable request.
