# Supplementary material for: EZH2 enhances PCV2 replication through inhibition of MMP1 and MMP12 transcription activity
Source: Vet Res. 2026 Jun 8;57:101. doi: 10.1186/s13567-026-01773-3 (PMC13244947; doi:10.1186/s13567-026-01773-3)
Supplement: Supplementary file 1 — Additional file 1. SiRNAs sequences. [file 13567_2026_1773_MOESM1_ESM.docx]

Additional file 1. siRNAs sequences

| EZH2-Sus-1004 | GCUCUGGACAACAAACCUUTT | AAGGUUUGUUGUCCAGAGCTT |
| --- | --- | --- |
| EZH2-Sus-1278 | CCAGUUCUUCAGAAGCAAATT | UUUGCUUCUGAAGAACUGGTT |
